# Comparison of hourly aerosol retrievals from JAXA Himawari/AHI in version 3.0 and a simple customized method

**DOI:** 10.1038/s41598-020-77948-5

**Published:** 2020-11-30

**Authors:** Weiwei Xu, Wei Wang, Biyan Chen

**Affiliations:** School of Geoscience and Info-Physics, Central South University, Changsha, China

**Keywords:** Climate sciences, Environmental sciences

## Abstract

Advanced Himawari imager (AHI) carried on the new-generation geostationary meteorological Himawari-8 satellite of Japan has been generating aerosol observations with a high temporal resolution since 7 July 2015. However, the previous studies lack a comprehensive quality assessment and spatial coverage analysis of AHI hourly aerosol products (level 3 version 3.0) across the full disk scan. The monitoring accuracy of different AHI aerosol products (AOD_pure_ and AOD_merged_) and a simple customized product (AOD_mean_) was evaluated against Aerosol Robotic Network (AERONET) and Maritime Aerosol Network (MAN) observations from May 2016 to February 2019 in this study. Results showed that AHI AOD_mean_ demonstrates a better agreement to AERONET AOD measurements than AOD_pure_ and AOD_merged_ over land (R = 0.81, bias =  − 0.011) and all the AHI land retrievals present a significant regional performance differences, while the relatively better performance is observed in AOD_merged_ over the coastal regions (R = 0.89, bias = 0.053). Over ocean, AHI exhibited overall overestimation in retrieving AOD against MAN observations and the relatively lower uncertainties were found in AOD_pure_ retrievals (R = 0.96, bias = 0.057). The hourly comparisons in different AHI products demonstrated a robust performance in the late afternoon (16:00–17:00 LT) over land and around the noon (10:00–13:00 LT) over coast. AHI AOD products indicated an obvious underestimation when compared to MODIS AOD retrievals over both land and ocean. Furthermore, the performance differences of AHI AOD products have also affected by the vegetation cover, pollution levels and relative humidity. For spatiotemporal coverage, the results of different AHI products demonstrated that AOD_mean_ can achieve relatively higher coverage than AOD_pure_ and AOD_merged_, and AHI retrievals present significant regional differences in coverage capability.

## Introduction

As an essential component of Earth’s climate system, aerosols are fine particles of solid or liquid that can suspend in the air^1–3^. Aerosols originate from natural (i.e., mineral dust, fog, sea salt, volcanic ash and biogenic emissions) or anthropogenic (i.e., haze, particulate air pollutants and biomass burning) activities and affect the radiation budget of the earth by direct and indirect radiative forcing^4–6^. High aerosol concentrations can also influence environmental quality and global climate^7,8^, degrade visibility^9^ and severely harm public health^10,11^. However, aerosol characteristics remain undefined due to its inhomogeneous composition, short lifespan and spatiotemporal variability^12,13^.

The Lidar known as an active remote sensing technology has been used to obtain atmospheric properties^14^, chlorophyll concentration^15^, leaf nitrogen concentration^16^, etc. However, passive satellite remote sensing has been more extensively utilized in the regional and global monitoring of aerosol optical properties and parameters, including Aerosol Optical Depth (AOD) and Angstrom Exponent (AE). Global AOD at different spatial and temporal resolutions is obtained using various satellite instruments. Atmospheric aerosol studies have applied satellite sensors such as the Total Ozone Mapping Spectrometer^17^, Polarization and Directionality of Earth Reflectances^18^, Medium Resolution Imaging Spectrometer^19^, Visible Infrared Imaging Radiometer Suite^20,21^, Ozone Monitoring Instrument^22^, Advanced Very High Resolution Radiometer^23^, Multi-angle Imaging SpectroRadiometer^24^, and Moderate Resolution Imaging Spectroradiometer (MODIS)^25^ are widely applied in atmospheric aerosol studies. On 18 December 1999 and 4 May 2002, the Terra and Aqua satellites of the Earth Observation System (EOS) were respectively launched into the earth’s orbit. The MODIS atmosphere team released the new MODIS Collection 6.1 (C6.1) product, which provides Dark Target (DT) AOD products for densely vegetated areas and Deep Blue (DB) AOD products for bright surfaces. Moreover, compared with the previous C6 retrievals against ground-truth AOD observations from Aerosol Robotic Network (AERONET), assessment results indicate that MODIS C6.1 aerosol products exhibit enhanced performance^26–28^. Although the polar or low orbit satellite measurements have provided AOD with high quality for global coverage, these detections are limited by the low temporal resolution.

On October 7, 2014, a latest generation of geostationary weather satellite called Himawari-8 operated by the Japan Meteorological Agency (JMA) was launched and carried with a primary sensor, the Advanced Himawari Imager (AHI). The frequency observations of Himawari-8 AHI have a potentiality to promote the identification and tracking of real-time dynamic aerosol properties over Asia–Pacific. Himawari-8 AHI released AHI level 2 (L2) aerosol products every 10 min on the basis of the common retrieval algorithm proposed by Yoshida et al.^29^; this algorithm contains AOD at 500 nm and AE between 400 and 600 nm with a spatial resolution of 0.05°. The hourly-combined algorithm with rigorous cloud-screening from L2 retrievals to improve the performance of AHI aerosol products was developed by Kikuchi et al.^30^. The main conclusions about the evaluation results of Himawari-8/AHI L2 aerosol product can be summarized as follows: (1) AHI retrievals showed relatively larger errors over the land and its performance was affected by the surface types, seasons, aerosol types and geometrical conditions; (2) The oceanic AHI AOD retrievals exhibited an overall overestimation; (3) AHI AE retrievals showed large estimation uncertainties over land, while the AHI coast AE agreed better with AERONET AE; (4) The AHI AOD demonstrated a worse performance compared to that of MODIS retrievals^31–34^. As for the latest version, Wang et al.^35^ found that the AHI Level 3 (L3) hourly AOD retrievals were generally fit well with AERONET measurements in Beijing, but still with a slight underestimation. Li et al.^36^ concluded that the AHI level 3 version 3.0 hourly aerosol products presented a better agreement with AERONET than previous two versions (1.0 and 2.0) over eastern China. Zhang et al.^37^ validated the performance of AHI L3 AOD_merged_ against AERONET AOD over land and revealed the regional and seasonal difference of AHI retrievals. The comparison of AHI hourly AOD products and MODIS C6.1 DB AOD products over China with AERONET AOD measurements indicated that MODIS retrievals demonstrated superior performance to that of AHI^38^. However, these studies about the newly updated AHI L3 aerosol products mostly focus on the partial regions or land areas and a comprehensive evaluation considering more influencing factors has not been conducted over the entire measuring region of Himawari-8. Furthermore, the performance differences and spatial coverage capacity of different Himawari-8/AHI hourly aerosol datasets have not yet been clarified in previous studies.

Therefore, this study aims to provide a thorough evaluation of different AHI AOD products and a simple customized product over ocean and land, thereby providing guidance for data users and improved direction of AHI aerosol retrieval algorithm for further researches. The paper is organized by the following structures. “Materials and methods” section reports the methods and datasets used to conduct the evaluation. “Results and discussion” section presents the comprehensive inter-comparison results of AHI aerosol retrievals. “Conclusions” section provides the summary and conclusions of this paper.

## Materials and methods

### Materials

**Figure 1:**
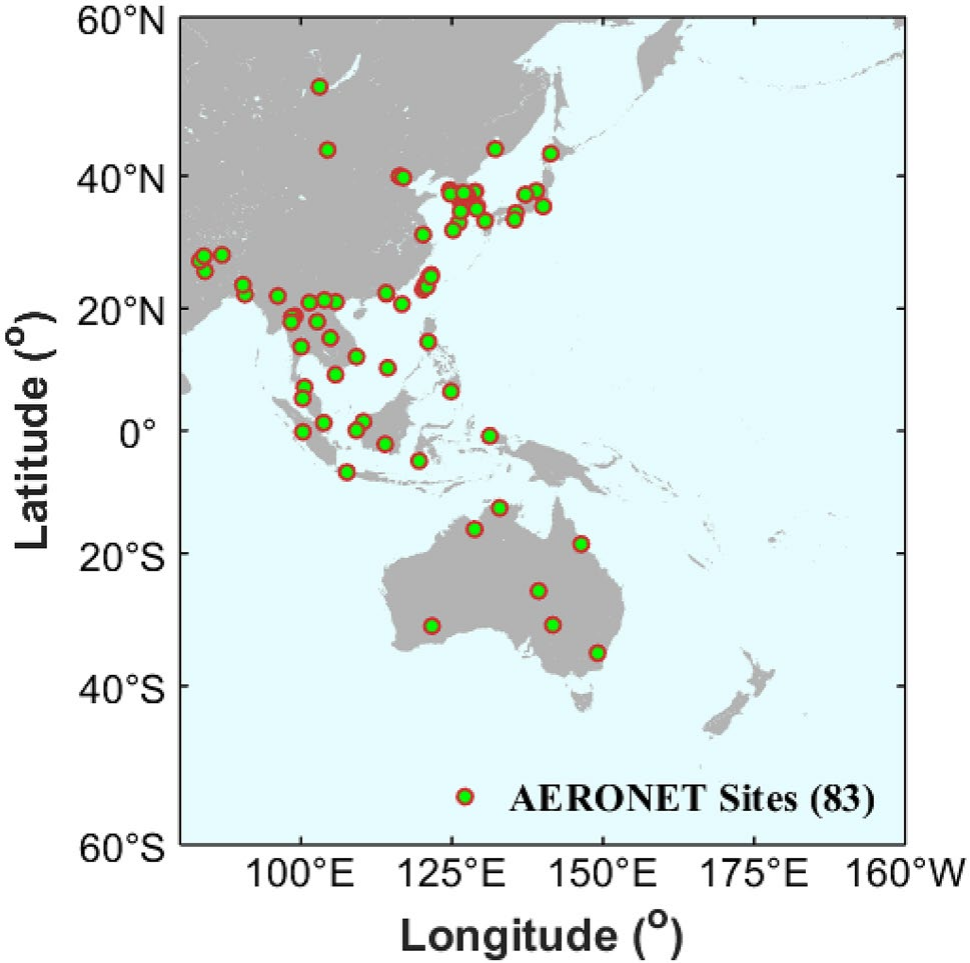
Study region and AERONET sites. The figure is generated by the software package M_Map^39^ (version 1.4 m; www.eoas.ubc.ca/~rich/map.html).

High-accuracy AERONET and MAN AOD observations are selected for validation and comparison to assess the newly released AHI L3 aerosol products over the full disk scans. The AHI aerosol products (level 2 version 2.1 and level 3 version 3.0), AERONET cloud-screened and quality-assured AOD (level 2.0 version 3) and all points MAN AOD (level 2) were collected from May 2016 to February 2019. Meanwhile, the MODIS C6.1 AOD products (Level 2) which are extensively adopted for the characterization of aerosol distribution and temporal variation were downloaded for comparison within the same period. To evaluate AHI AOD products under different meteorological factors, the relevant meteorological datasets were also collected in this study. The fine particulate matter (PM_2.5_) observations of major cities in China were downloaded from the China Environmental Monitoring Center (CNEMC) (http://www.cnemc.cn/), and the relative humidity (RH) and planetary boundary layer height (PBLH) were obtained from the ERA5 reanalysis dataset of European Centre for Medium‐Range Weather Forecasts (ECMWF) (https://www.ecmwf.int/en/forecasts/datasets/reanalysis-datasets/era5). The distribution of 86 AERONET sites in the full scene of AHI measurement (80°E–160°W and 60°N–60°S) is shown in Fig. 1.

#### AHI hourly aerosol product

Two primary datasets known as AOD_pure_ and AOD_merged_ are included in the newly updated AHI hourly aerosol products provided by the Japan Aerospace Exploration Agency (JAXA). The strict cloud-screening quality control of L2 retrievals is applied to reduce cloud contamination in AOD_pure_ and AOD_merged_ is obtained from the spatiotemporal optimum interpolation of AOD_pure_ within one hour to compensate for the missing values due to the elimination of sensor noise and insufficient cloud-screening in the AOD_pure_ product^29,30^. Four confidence levels, namely, “very good”, “good”, “marginal” and “no confidence” (or “no retrieval”), are included in the quality assurance (QA) flag of AOD_pure_ and AOD_merged_, and only the “very good” level of pure and merged AOD observations were used. More details of the AHI L3 aerosol product scheme is available in the official documentation instruction^40^. Moreover, we have used a customized method to derive the average AOD and AE over an hour (denoted by AOD_mean_ and AE_mean_) based on L2 retrievals using the same QA level (“very good”) for quality control to perform a more extensive evaluation in this study.

Matching AERONET observations with AHI retrievals in the same spatiotemporal scale is the first step to evaluate the performance of hourly AOD products over land and ocean. Considering the AHI AOD retrievals estimated with different temporal definitions, the corresponding matching methods were formulated: (1) The median observation time of L2 retrievals is adopted in AHI AOD_mean_ and its estimates within ± 30 min of AERONET AOD observations were collected in this study; (2) AHI AOD_pure_ is the available values after threshold filtering in L2 retrievals, thereby the temporal window of AOD_pure_ was set as ± 10 min; (3) AHI AOD_merged_ is derived from 6 slots of AOD_pure_ (ie., past 1 h) with the weighted-average interpolation. Consequently, the AERONET AOD observations within the past 1 h from AOD_merged_ were collocated. As for the spatial scale, AHI AOD observations within the radius of 15 km (3 × 3 pixels) centered on surrounding the ground-based sites were selected. The twice standard deviation check was executed to screen out those abnormal satellite AOD measurements within the domain before the calculation of its mean values^37^. Moreover, a reliable collocation requires at least 10 AHI AOD retrievals within the spatiotemporal window.

#### MODIS

The new MODIS (Terra/Aqua) C6.1 level 2 atmospheric aerosol products (MOD04_L2 for Terra and MYD04_L2 for Aqua) with a spatial resolution of 10 km were used in this study to gather the DT AOD retrievals from May 2016 to February 2019. The scientific data set (SDS) of MODIS C6.1 DT AOD retrievals named “Optical Depth Land And Ocean” were collected and such data were filtered with the recommended high-quality flag over land (QF = 3) and ocean (QF > 0)^25^. In addition, the MODIS AOD was transformed from 550 to 500 nm using the ground-measured AERONET AE. The ground-based network data were matched with MODIS aerosol product in time and location by using the following criteria: (1) a 30-min interval limitation of the AERONET and MODIS AOD; (2) a total of 15-km geographical distance between the MODIS AOD and ground site; (3) no fewer than 10 satellite AOD retrievals in the matching range. The variance check was also carried out in MODIS AOD data as AHI collocations. In addition, Normalized difference vegetation index (NDVI) is the commonly used measurements of surface vegetation dynamics in most studies, and the data was also acquired from MODIS 16-day composite NDVI products (MOD13C1/MYD13C1) in this study.

#### AERONET

The AERONET is a worldwide ground-based sunphotometer monitoring network that provides prolonged, continuous and high-accuracy measurements of the atmospheric aerosol parameters^41^. Available AERONET AOD data online have the following three quality levels: Level 1.0 (unscreened), Level 1.5 (cloud cleared), and Level 2.0 (cloud cleared and quality controlled)^42,43^. The AHI AOD performance was evaluated using the recommended AERONET version 3 observations (the estimated uncertainty from ~ 0.01 to 0.02) with a fully automatic cloud screening and quality control in this work^44^. The AERONET AOD (version 3 level 2.0) has a high temporal frequency of approximately 3–15 min for the following bands: 340, 380, 440, 500, 675, 870, 1020, and 1640 nm^44^, and the measurements at 500 nm channel was employed for the inter-comparison of satellite-based AOD observations. Additionally, the characteristics of sand-water mixture surface and complex aerosol types in the coastal regions are widely different from the inland aerosol properties^45,46^. Therefore, the AERONET sites in this study were divided into inland and coastal measurements.

#### MAN

The MAN is a ship-based network established as a complement to AERONET, and has been collecting AOD observations across oceans worldwide by using Microtops II handheld Sun photometers^47^. MAN has been providing high-quality aerosol observations with an estimated uncertainty of <  ± 0.02, which is sufficient for the evaluation of satellite observations over oceans^47,48^. Marine aerosols are measured by the Microtops II instruments in 5 bands within the scope of 340 nm to 1020 nm^47^. In particular, MAN AOD measurements at 500 nm were selected for the collocation and evaluation in this study. The average of AHI AOD within the various spatiotemporal window centered on a MAN observation site was also used as a matching value in this work. Similarly, at least 10 AHI AOD retrievals within the matching range were required in an eligible collocation of AHI and MAN.

### Evaluation criteria

The expected error (EE) envelope containing at least 68% (one standard deviation) of the matchups on a scatterplot was used in the subsequent validation with AERONET to assess the quality and uncertainty of satellite retrievals quantitatively. Specifically, the expected accuracy (EA) and expected precision (EP) were separated to describe the EE envelope of AHI more accurately, which represent the average deviations from truth and the fluctuation of deviations, respectively^32,49^. Thus, the EE can be expressed as EA ± EP. Moreover, the accuracy and performance of AHI AOD were analyzed by using the following statistical parameters: (1) bias, representing the average error between the different data sets (AHI–AERONET); (2) root mean square error (RMSE), the standard deviation of the prediction errors; (3) correlation coefficient (R), the statistical standard to evaluate the correlation between retrievals and ground-based measurements; (4) global climate observing system fraction (GCOSF), the fraction of satellite retrievals that meet the Global Climate Observing System requirement for AOD accuracy (smaller than the maximum of 0.03 or 10%)^32,50^; (5) mean percentage error, the bias of matched satellite retrievals from AERONET sites, which can be calculated by ${\text{mean}}(\frac{{{\text{AHI}}_{{{\text{AOD}}}}  - {\text{AERONET}}_{{{\text{AOD}}}} }}{{{\text{AERONET}}_{{{\text{AOD}}}} }}) \times 100\%$.

## Results and discussion

### Regional evaluation with AERONET

**Figure 2:**
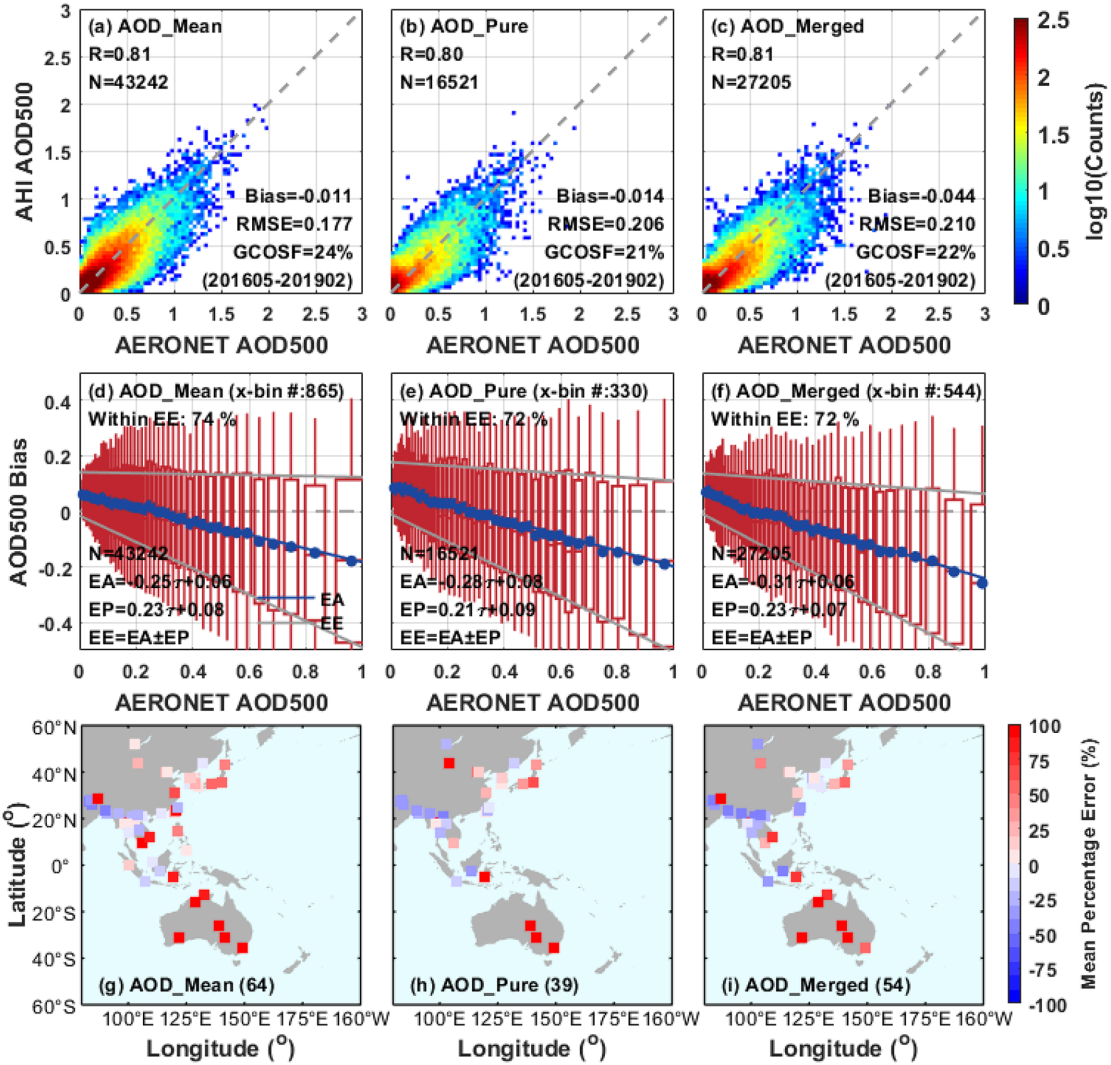
Validation of AHI AOD aerosol retrievals with AERONET over land from May 2016 to February 2019. (**a**–**c**) Density scatter plots of AHI AOD retrievals versus AERONET observations. (**d**–**f**) Box plots of AOD bias (AHI AOD-AERONET AOD) against AERONET observations. The blue dots, red center lines, and red boxes in the vertical of these panels indicate the means, medians, and 1 standard deviation of the AOD differences, respectively. The linear regressions to mean biases (blue solid circles), i.e. expected accuracy (EA), are denoted by the blue line. Meanwhile, the linear regressions of the 1 standard deviation of the biases, i.e. the expected precision (EP), are denoted by the upper and lower limits of the red boxes. The two grey lines represent the expected error (EE) calculated by the formula of EA ± EP. (**g**–**i**) Mean percentage error of AHI AOD retrievals at each AERONET site. The validation results of different AHI L3 AOD datasets (AOD_mean_, AOD_pure_ and AOD_merged_) are listed in columns 1, 2 and 3, respectively. The figure is generated by the software package M_Map^39^ (version 1.4 m; www.eoas.ubc.ca/~rich/map.html).

As shown in the scatter plots of Fig. 2a–c (first row), the performance of AHI AOD_pure_ and AOD_merged_ retrievals is similar over land, with the commensurate R (0.80 vs. 0.81) and GCOSF (21% vs. 22%), suggesting the original retrieval accuracy of AHI AOD_pure_ is nearly maintained in the spatiotemporal interpolation of AOD_merged_. Almost the same performance of AOD_pure_ and AOD_merged_ is also mentioned in Li et al.^36^. Moreover, AOD_mean_ shows a slightly better agreement with AERONET retrievals when compare it to that of AOD_pure_ and AODmerged, with an increased R of 0.81, a larger GCOSF of 24%, a smaller bias of − 0.011, and a lower RMSE of 0.177. It is equally remarkable that a larger number of collocations are observed in AOD_mean_ than it of AOD_merged_ with a spatiotemporal window of the same size (43,242 vs. 27,205).

Figure 2d–f (second row) show the boxplots of AHI AOD biases with the variation of AERONET AOD. According to the statistical results, the mean AHI AOD biases change from positive to negative with the increase of ground-truth AOD, and the minimum deviations can be found when the AERONET AOD is between 0.2 and 0.3. The AHI AOD biases as a function of AERONET AOD are described by EA: EA_mean_ =  − 0.25 × τ_AERONET_ + 0.06, EA_pure_ =  − 0.28 × τ_AERONET_ + 0.08 and EA_merged_ =  − 0.31 × τ_AERONET_ + 0.06. The systematic underestimation trend of AHI AOD can be found in other studies^36–38^ and it could be attributed to the overestimation of surface reflectance estimation and limited representation of aerosol model^30,32^. The EEs of different AHI AOD retrievals are shown in Fig. 2d–f: [− 0.48 × τ_AERONET_ − 0.02; − 0.02 × τ_AERONET_ + 0.14], [− 0.49 × τ_AERONET_ − 0.01; − 0.07 × τ_AERONET_ + 0.17] and [− 0.54 × τ_AERONET_ − 0.01; − 0.08 × τ_AERONET_ + 0.13], and there are 74%, 72% and 72% of paired retrievals falling within in the envelopes, respectively.

The validation results in Fig. 2g–i (third row) present the AOD mean percentage error of three AHI datasets over different land regions. Significant positive biases and negative biases of AHI AOD are still noticed over Australia and Southeast Asia in most of the AERONET stations respectively, which have been found in the previous AHI L2 aerosol products^31–33^ and the validation results of AHI L3 aerosol products^37^. Comparatively, the AHI AOD performs better over eastern China, Korean Peninsula and Japan with a relatively lower uncertainty. These regional differences of AHI aerosol retrievals over land may be related to the surface types (e.g. dark or bright surface) and aerosol models (e.g. sphericity or size distribution), which suggests that the AHI retrieval algorithm and performance need to be further improved and perfected for such areas. As for the performance of different AHI AOD products, AOD_pure_ and AOD_merged_ tend to be slightly better consistent with AERONET AOD than AOD_mean_ over eastern China, Korean Peninsula and Japan, while the AOD_mean_ retrievals exhibit smaller biases in many Southeast Asia sites.

**Figure 3:**
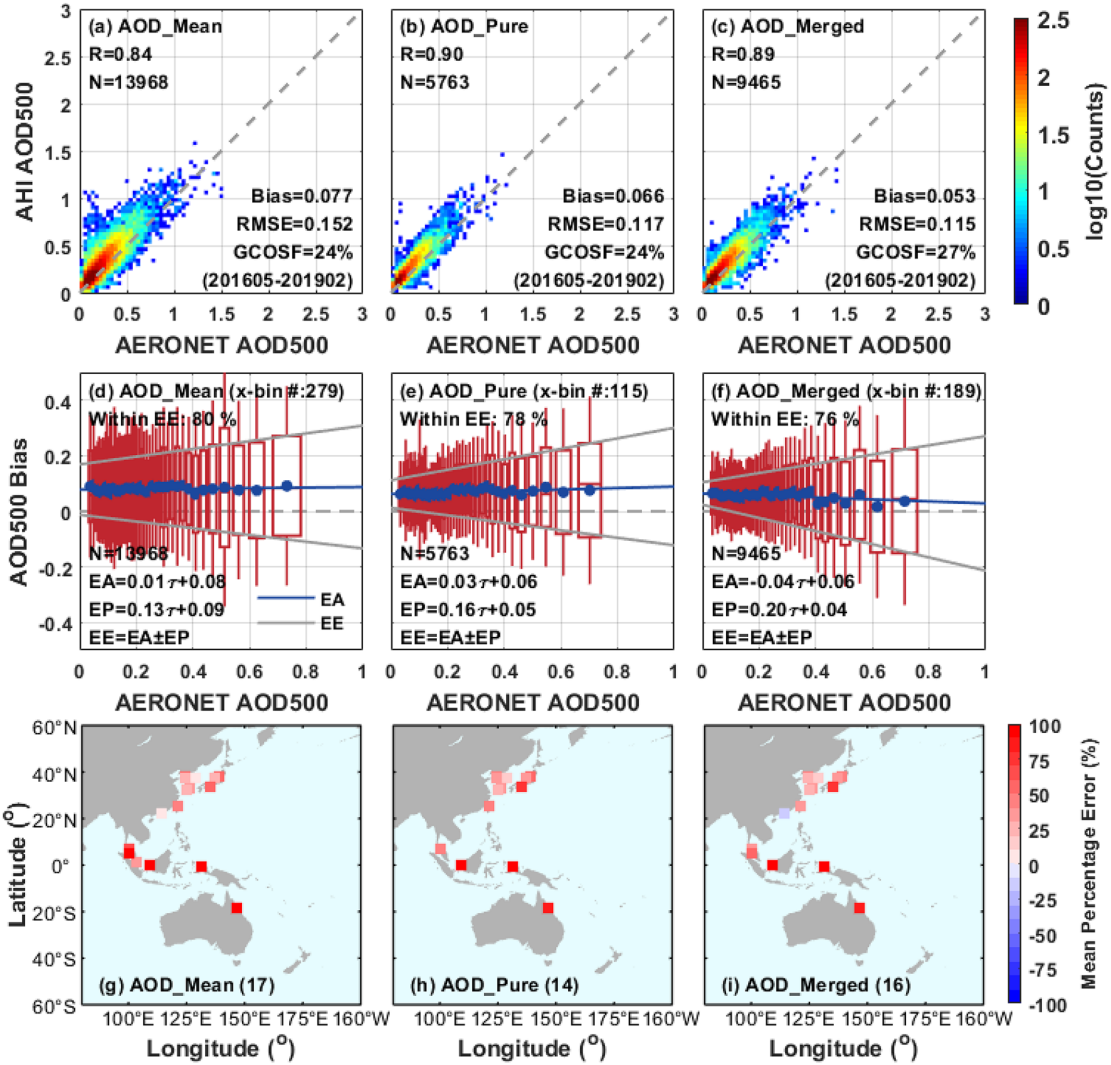
Validation of AHI AOD aerosol retrievals with AERONET over coast from May 2016 to February 2019. (**a**–**c**) Density scatter plots of AHI AOD retrievals versus AERONET observations. (**d**–**f**) Box plots of AOD bias (AHI AOD-AERONET AOD) against AERONET observations. The blue dots, red center lines, and red boxes in the vertical of these panels indicate the means, medians, and 1 standard deviation of the AOD differences, respectively. The linear regression to mean biases (blue solid circles), i.e. expected accuracy (EA), are denoted by the blue line. Meanwhile, the linear regressions of the 1 standard deviation of the biases, i.e. the expected precision (EP), are denoted by the upper and lower limits of the red boxes. The two grey lines represent the expected error (EE) calculated by the formula of EA ± EP. (**g**–**i**) Mean percentage error of AHI AOD retrievals at each AERONET site. The validation results of different AHI L3 AOD datasets (AOD_mean_, AOD_pure_ and AOD_merged_) are listed in columns 1, 2 and 3, respectively. The figure is generated by the software package M_Map^39^ (version 1.4 m; www.eoas.ubc.ca/~rich/map.html).

The comparison for the AHI L3 AOD products against coastal AERONET sites is shown in Fig. 3. The first row presents the scatter plots for AHI AOD_mean_, AOD_pure_ and AOD_merged_ products versus AERONET AOD over coastal regions from May 2016 to February 2019, respectively. In comparison to the land aerosol retrieval analysis presented above, the coastal AOD retrievals from AHI referenced to AERONET have a relatively better performance with a higher R of 0.84–0.90, and a smaller RMSE of 0.115–0.152 than that of land. The comparison results show that the AOD_merged_ has a higher AOD retrieval accuracy than AOD_mean_ and AOD_pure_ over coastal areas, with an increased R (0.89 vs. 0.84 and 0.90), a larger GCOSF (27% vs. 24% and 24%), a smaller bias (0.053 vs. 0.077 and 0.66), and a lower RMSE (0.115 vs. 0.152 and 0.117). Moreover, smaller estimation biases are also found in AOD_pure_ compared to AOD_mean_ retrievals (bias = 0.066 vs. 0.077; RMSE = 0.117 vs. 0.152).

Figure 3d–f (second row) show the differences between AHI aerosol products and ground-based AOD from May 2016 to February 2019. For coastal areas, the linear regression for these positive differences of AHI AOD_mean_ in Fig. 3d is represented by the EA = 0.01 × τ_AERONET_ + 0.08, which demonstrates a very weak dependence between the AHI-AERONET AOD and AERONET AOD than that of land. Figure 3e shows that the trend of AHI AOD_pure_ biases relative to AERONET is analogous to that of AOD_mean_ in Fig. 3d over coastal areas, with similar EA = 0.03 × τ_AERONET_ + 0.06. However, the AOD_merged_ slightly declined with the increase of AERONET AOD and the linear relationship is represented by the equation of EA =  − 0.04 × τ_AERONET_ + 0.06. The EEs of different AHI AOD retrievals over coastal regions in Fig. 3d–f are listed as follows: [− 0.12 × τ_AERONET_ – 0.01; 0.14 × τ_AERONET_ + 0.17], [− 0.13 × τ_AERONET_ − 0.01; 0.19 × τ_AERONET_ + 0.11] and [− 0.24 × τ_AERONET_ + 0.02; 0.16 × τ_AERONET_ + 0.10], and 80%, 78% and 76% of the matchups are falling in the range, respectively.

Figure 3g–i (third row) show the comparisons of regional AOD mean percentage error between different AHI retrieval datasets and ground measurements for each coastal AERONET site from May 2016 to February 2019. The overall mean biases of L3 AHI-derived aerosol retrievals against AERONET present positive over coastal regions and extremely high biases can be found in the AERONET sites located on the Australian coast and near the equator. Notably, the AERONET AOD of Hong Kong (114.180°E, 22.303°N) in southern China with a smallest mean positive biases in AOD_mean_ (0.005) and a special mean negative bias (− 0.120) in AOD_merged_, which may be influenced by the dominate urban fine aerosols (moderately absorbing) from the industrialized regions of Pearl River Delta^32,51^. Overall, the three datasets perform a similar distribution of mean biases against the coastal AERONET sites.

**Figure 4:**
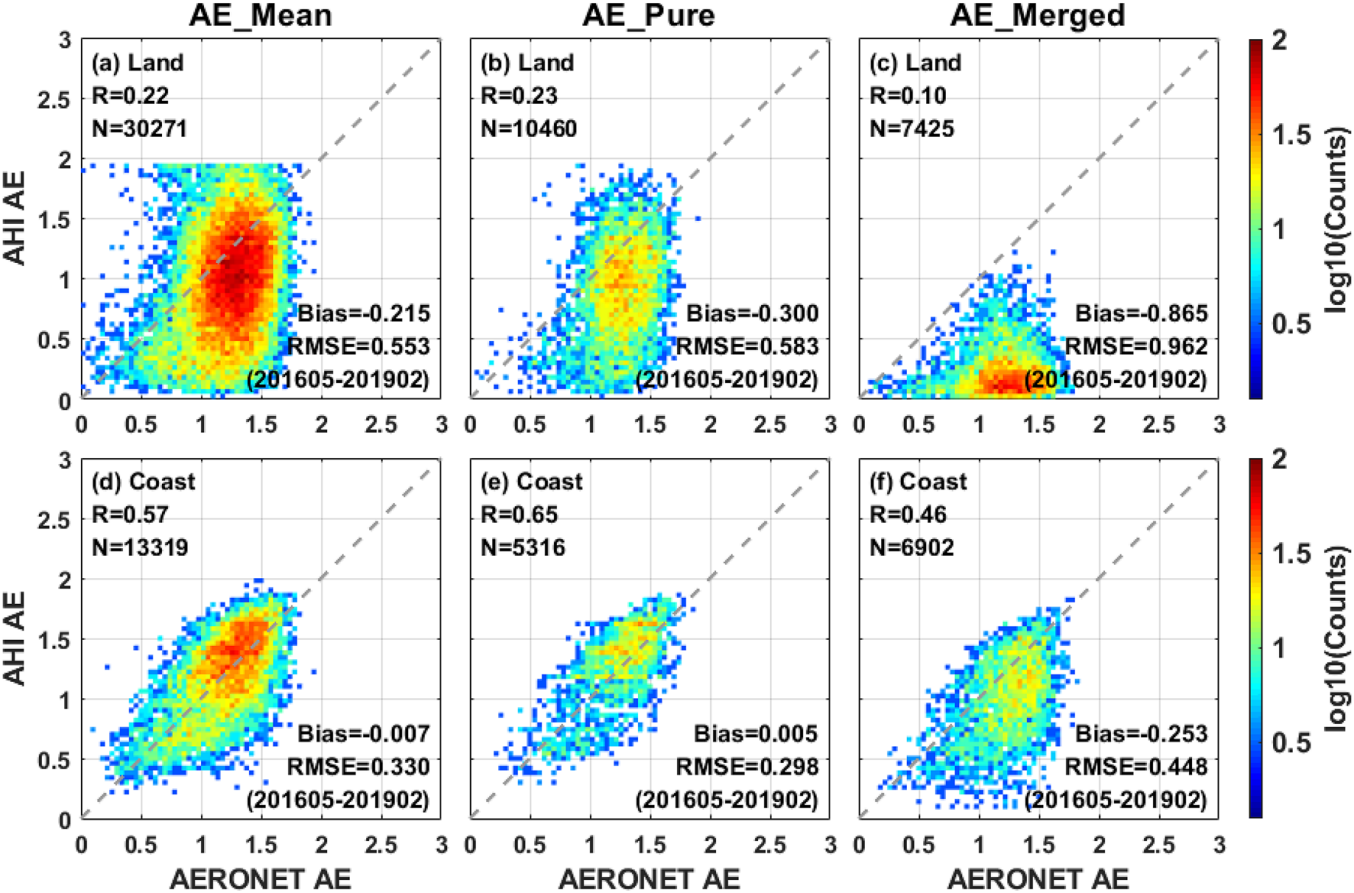
Validation of AHI AE retrievals with AERONET over land and coast from May 2016 to February 2019. (**a**–**c**) Density scatter plots of AHI AE versus AERONET measurements over land. (**d**–**f**) Density scatter plots of AHI AE versus AERONET measurements over coast. The validation results of different AHI L3 AE datasets (AE_mean_, AE_pure_ and AE_merged_) are listed in columns 1, 2 and 3, respectively.

The different AHI AE retrievals versus ground measurements over both land and coast are compared in the scatter plots of Fig. 4. The comparative results reveal that the AHI AE performance over land is poorer than the retrievals over coast. The land AE retrievals in first row show the worse performance of AE_merged_ with larger estimation uncertainty (bias =  − 0.865, RMSE = 0.962) compared to that of AE_mean_ and AE_pure_ (bias =  − 0.215 and − 0.300, RMSE = 0.553 and 0.583). By contrast, there is a relatively better AE retrieval performance over coast in Fig. 4d–f with smaller estimation uncertainty (bias =  − 0.253–0.005, RMSE = 0.298–0.448), but an obvious underestimation can still be found in AE_merged_ retrievals (bias =  − 0.253, RMSE = 0.448). However, AHI AE_pure_ is adopted from the L2 AE retrievals when AOD_pure_ is a valid value^29^, and it shows a similar agreement against AERONET AE with AE_mean_ retrievals over both land and coast in Fig. 4. In addition, the coast AE_merged_ retrievals present a relatively smaller bias than the land AE_merged_ (bias =  − 0.253 vs. bias =  − 0.865). Overall, the poor performance of AHI L2 AE retrievals^31–33^ continues to be noted in the L3 hourly products.

In AHI L3 hourly combined algorithm, the AE_merged_ product was obtained from AE_pure_ retrievals by the same optimal interpolation method as AOD_merged_ and can be calculated as follows^29,30^:

1 $$ AE_{merged} (x_{0} ,y_{0} ,t_{0} ) = \sum\limits_{i = 0}^{N} {W_{i} AE_{pure} (x_{i} ,y_{i} ,t_{i} )} $$

2 $$ W_{i} = \frac{{\frac{1}{{\sigma_{pure} (x_{i} ,y_{i} ,t_{i} )^{2} }}}}{{\frac{1}{{\sigma_{merged} (x_{0} ,y_{0} ,t_{0} )^{2} }}}} $$

3 $$ \frac{1}{{\sigma_{merged} (x_{0} ,y_{0} ,t_{0} )^{2} }} = \mathop \sum \limits_{i = 0}^{N} \frac{1}{{\sigma_{pure} (x_{i} ,y_{i} ,t_{i} )^{2} }} $$

where $AE_{pure} (x_{i} ,y_{i} ,t_{i} )$ is the AE_pure_ retrievals at a given time t_i_ and location (x_i_, y_i_) within a radius of 12.5 km and past 1 h from (x_0_, y_0_, t_0_). *N* is the total number of valid pixels in the calculation domain. $\sigma_{pure} (x_{i} ,y_{i} ,t_{i} )^{2}$ and $\sigma_{merged} (x_{0} ,y_{0} ,t_{0} )^{2}$ are the error variance. As shown in Eq. (1), the calculation of AE_merged_ depends on AE_pure_ retrievals and weight coefficient *W*_*i*_, but AE_pure_ has been proved to have reliable accuracy in Fig. 4b,e. Further, $\sigma_{pure} (x_{i} ,y_{i} ,t_{i} )^{2}$ is fundamentally derived from a lookup table that is calculated from the AOD spatiotemporal variability. Thus, the same weight as AOD_merged_ retrievals may result in the poor performance of the AE_merged_. Moreover, the aerosol spatiotemporal variability is larger over land than over ocean, which may lead to the AE_merged_ performance differences between land and coast in Fig. 4c,f.

**Figure 5:**
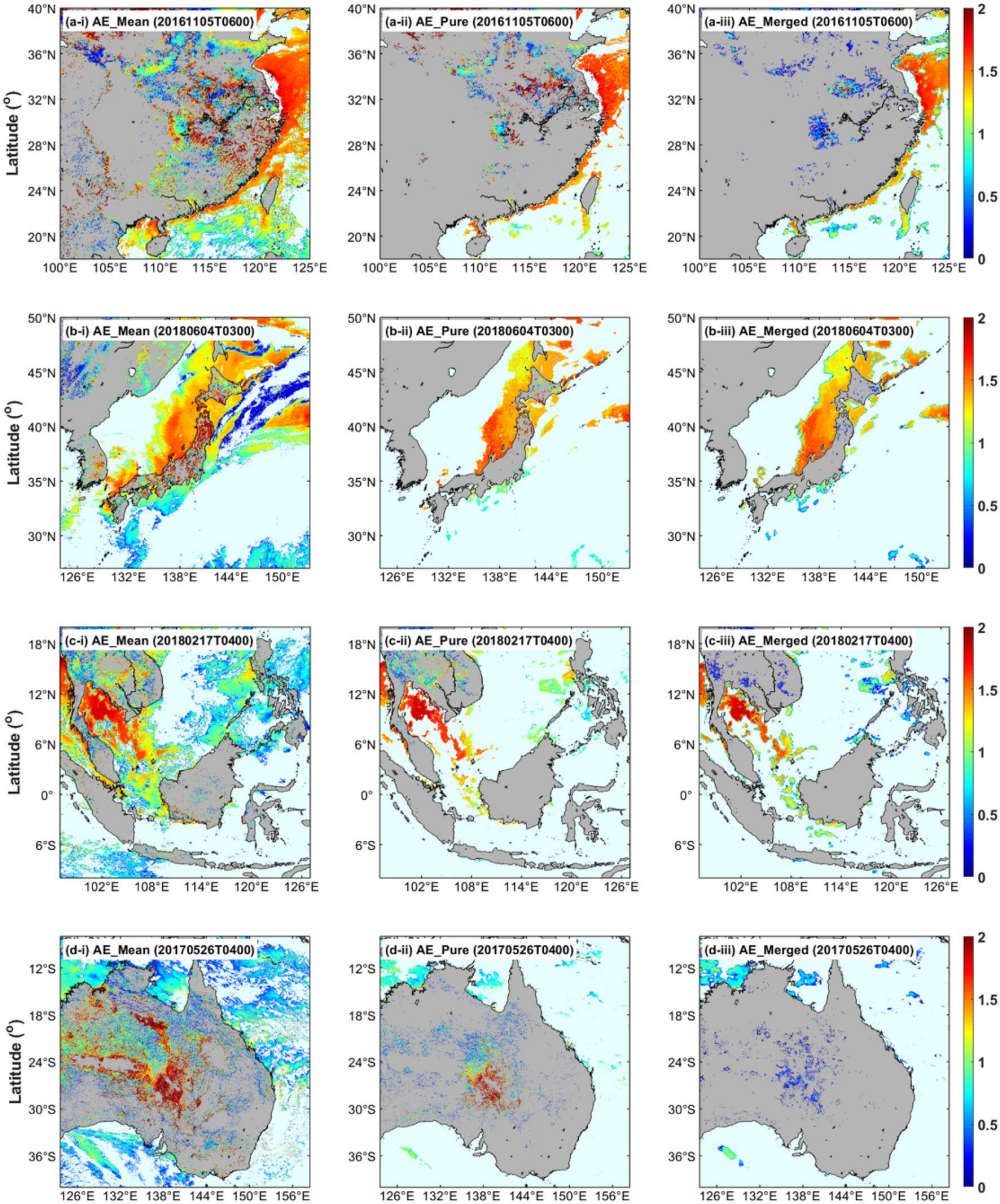
The AHI AE retrievals with QA screening (QA flag = very good) over the four sub-regions of southeastern China (**a**), Northeast Asia (**b**), Southeast Asia (**c**) and eastern Australia (**d**). The subplots (i–iii) denote different AHI products: (i) AE_mean_, (ii) AE_pure_ and (iii) AE_merged_. The figure is generated by the software package M_Map^39^ (version 1.4 m; www.eoas.ubc.ca/~rich/map.html).

In order to describe the performance differences of AHI products more intuitively, a display of regional AE retrievals is created in Fig. 5, and four regions (i.e., southeastern China, Northeast Asia, Southeast Asia and eastern Australia) within the AHI full disk coverage, including land and ocean, were mainly selected for this study. These samples in Fig. 5 were randomly selected when sufficient observations were available in the target region. Overall, the spatial coverage of AE_mean_ is substantially higher compared to AE_pure_ and AE_merged_ retrievals over both land and ocean. It should be pointed out that the AE_merged_ retrievals are universally lower than AE_mean_ and AE_pure_ in all regions over land, which is consistent with the results shown in Fig. 4a–c. Furthermore, AHI AE_merged_ retrievals present a relatively reliable performance over coast and ocean in the subplot (iii) of Fig. 5, although many low value anomalies can be found at the edge of retrieval areas. These findings confirm the defects in the AE_merged_ retrieval algorithm, but further investigation on underlying causes and improvement methods is required in future research.

### Hourly evaluation with AERONET

**Figure 6:**
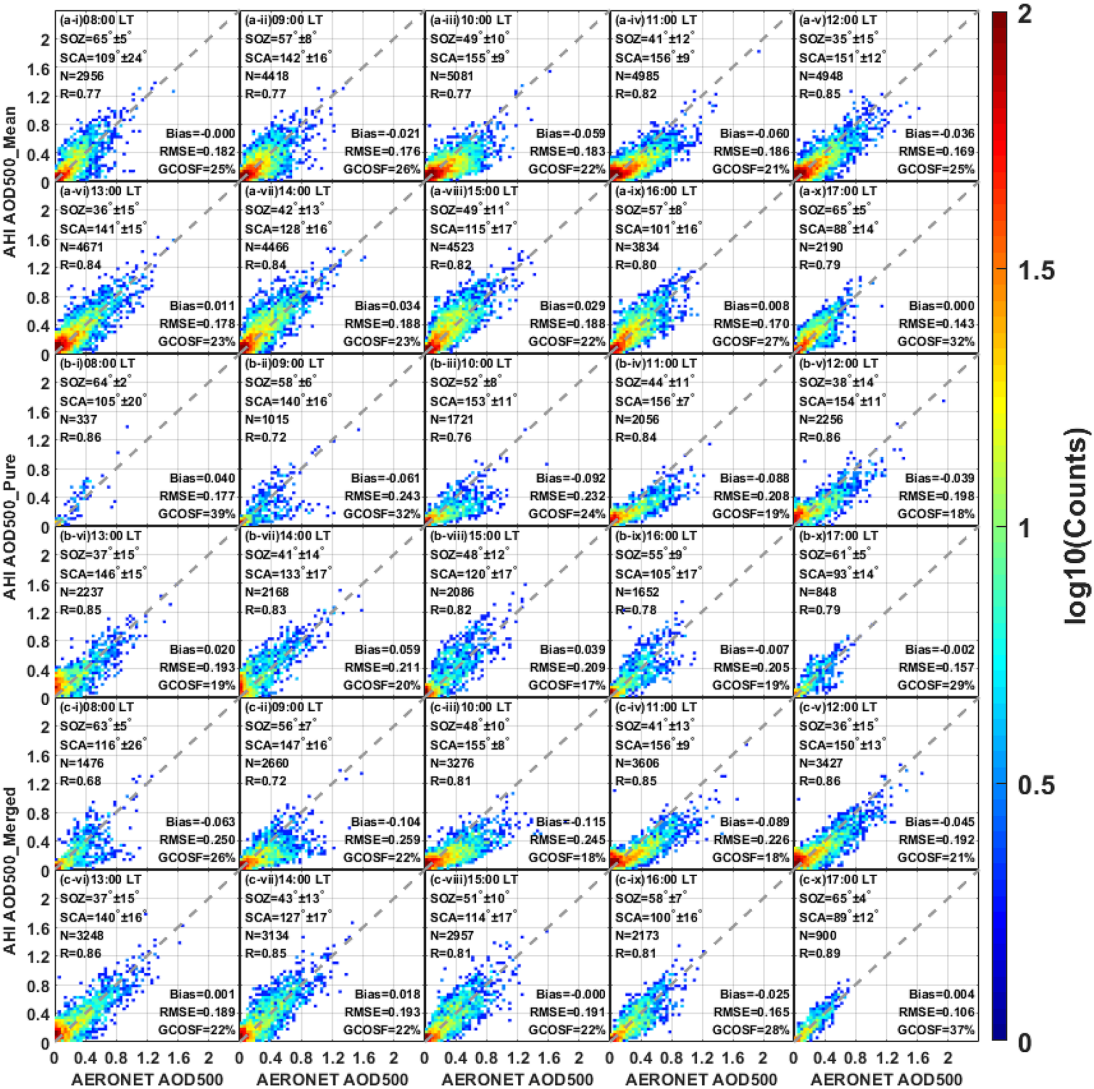
Scatter plots of AHI land AOD versus AERONET measurements during various daytime periods (local time). The panels (**a**–**c**) represent different AHI L3 AOD datasets (AOD_mean_, AOD_pure_ and AOD_merged_), respectively. The subplots (i–x) indicate the daytime hours from 8:00 LT–17:00 LT: (i) 08:00, (ii) 09:00, (iii) 10:00, (iv) 11:00, (v) 12:00, (vi) 13:00, (vii) 14:00, (viii) 15:00, (ix) 16:00 and (x) 17:00. SOZ = solar zenith angle; SCA = scattering angle.

It is reported in previous studies that the performance of AHI aerosol products exhibits an obvious variation at different daytime hours^31–33,37,38^. Accordingly, the evaluations of AHI temporal variation are also executed in this study. The local time (LT) of AHI measurements are translated from universal time coordinated (UTC) through the longitude of AERONET sites. The validation results of AHI L3 AOD datasets for different daytime hours (8:00–17:00 LT) over land are displayed in Fig. 6. The analysis indicates that the AOD_merged_ performs better than AOD_mean_ in the late afternoon (16:00–17:00 LT) but with more underestimations before 13:00 LT (bias =  − 0.115 to − 0.045 vs. bias =  − 0.060–0.000). These discrepancies may be result from the frequent occurrence of cloud-contaminated pixels in the afternoon^52^. By comparison with AOD_pure_, AHI AOD_merged_ retrievals are more consistent with ground-based measurements after 11:00 LT, but poorer performance can be found in the morning (8:00–11:00 LT). Overall, Himawari-8/AHI can more accurately obtain AOD retrievals at 16:00–17:00 LT than in other periods, which is probably related to the scattering angle. Furthermore, an obvious tendency of underestimating the aerosol loadings can be noticed in AHI AOD relative to AERONET AOD at 10:00–11:00 LT, with a large negative bias of − 0.115 to − 0.059 in different products.

**Figure 7:**
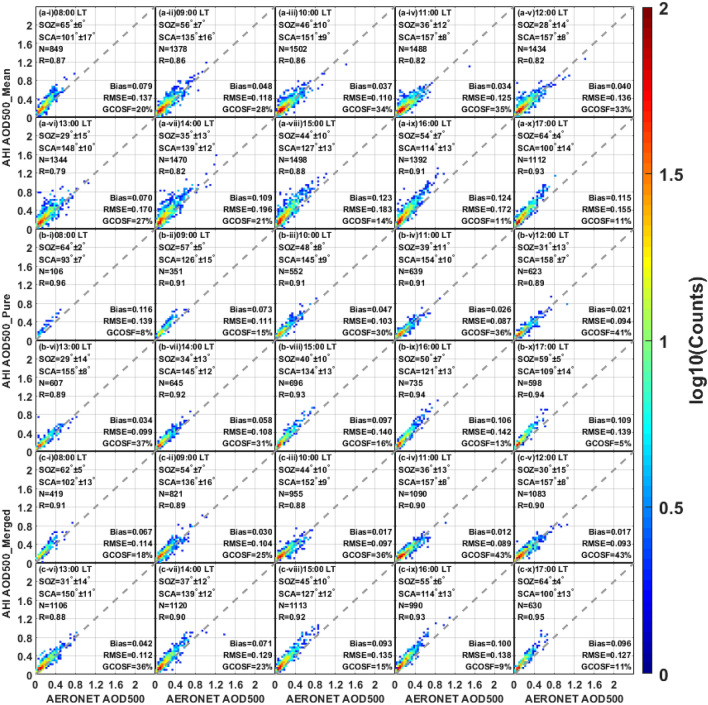
Scatter plots of AHI coast AOD versus AERONET observations during various daytime periods (local time). The panels (**a**–**c**) represent different AHI L3 AOD datasets (AOD_mean_, AOD_pure_ and AOD_merged_), respectively. The subplots (i–x) indicate the daytime hours from 8:00 LT–17:00 LT: (i) 08:00, (ii) 09:00, (iii) 10:00, (iv) 11:00, (v) 12:00, (vi) 13:00, (vii) 14:00, (viii) 15:00, (ix) 16:00 and (x) 17:00. SOZ = solar zenith angle; SCA = scattering angle.

By comparison with the land assessments, the hourly validation results of the AHI AOD retrievals against the ground-based AOD measurements over the coast are shown in Fig. 7. As compared to the AOD_mean_, AOD_merged_ collocations are generally fit better against AERONET AOD in each subplot, with a smaller bias (0.012–0.100 vs. 0.034–0.124), a lower RMSE (0.089–0.138 vs. 0.110–0.196) and a larger R (0.88–0.95 vs. 0.79–0.93), although slightly fewer retrievals fall within GCOSF at 8:00– 9:00 LT and 16:00 LT. In Fig. 7b,c, the AHI AOD_merged_ retrievals perform a little bit better than AOD_pure_ during most of the day, except for a large bias in the period of 13:00–14:00 LT. Overall, all the AHI products tend to overestimate the coastal AOD during the daytime, especially for the retrievals before 9:00 LT and after 13:00 LT. Moreover, AHI costal retrievals show the better performance at 10:00–13:00 LT than other times, with the relatively smaller bias (0.012–0.070), lower RMSE (0.087–0.170) and higher GCOSF (27–43%), which indicates that a smaller solar zenith angle or a large scattering angle may help to the high-quality coastal AOD retrieval.

**Figure 8:**
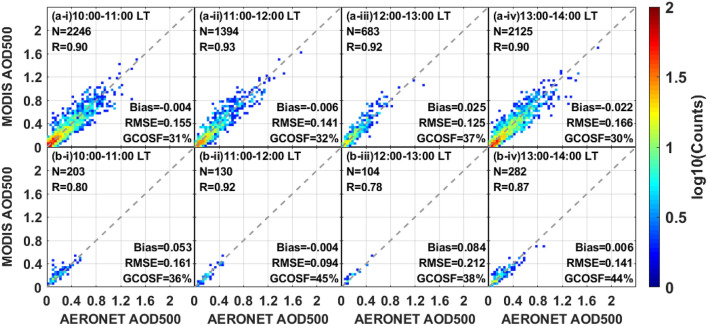
Scatter plots of MODIS AOD versus AERONET observations over land (**a**) and coast (**b**) during various daytime periods (local time). The subplots (i–iv) indicate the daytime hours from 10:00 LT to 14:00 LT: (i) 10:00–11:00, (ii) 11:00–12:00, (iii) 12:00–13:00 and (iv) 13:00–14:00.

To explore the temporal performance difference of AHI and MODIS aerosol retrievals, the hourly variations of MODIS AOD over both land and coast are presented in Fig. 8. Unlike the geostationary satellite, Terra-MODIS (ascending orbit) and Aqua-MODIS (ascending orbit) can only provide global observations at about 10:00–12:00 LT and 12:00–14:00 LT in the daytime, respectively. Compare to the AHI retrievals in Fig. 6, the performance of MODIS observations is comparatively better over land during the same period with 30–37% of retrievals falling within the GCOSF, larger R (0.90–0.92) and smaller RMSE (0.141–0.166). As for the coastal retrievals, the accuracy of MODIS AOD is similar to that of AHI AOD_merged_ before 12:00 LT (R = 0.80–0.92, within GCOSF = 36–45% vs. R = 0.88–0.90, within GCOSF = 36–43%), but all AHI products show a relatively larger bias after 13:00 LT (bias = 0.058–0.109 versus bias = 0.006).

### Evaluation for angular dependence

**Figure 9:**
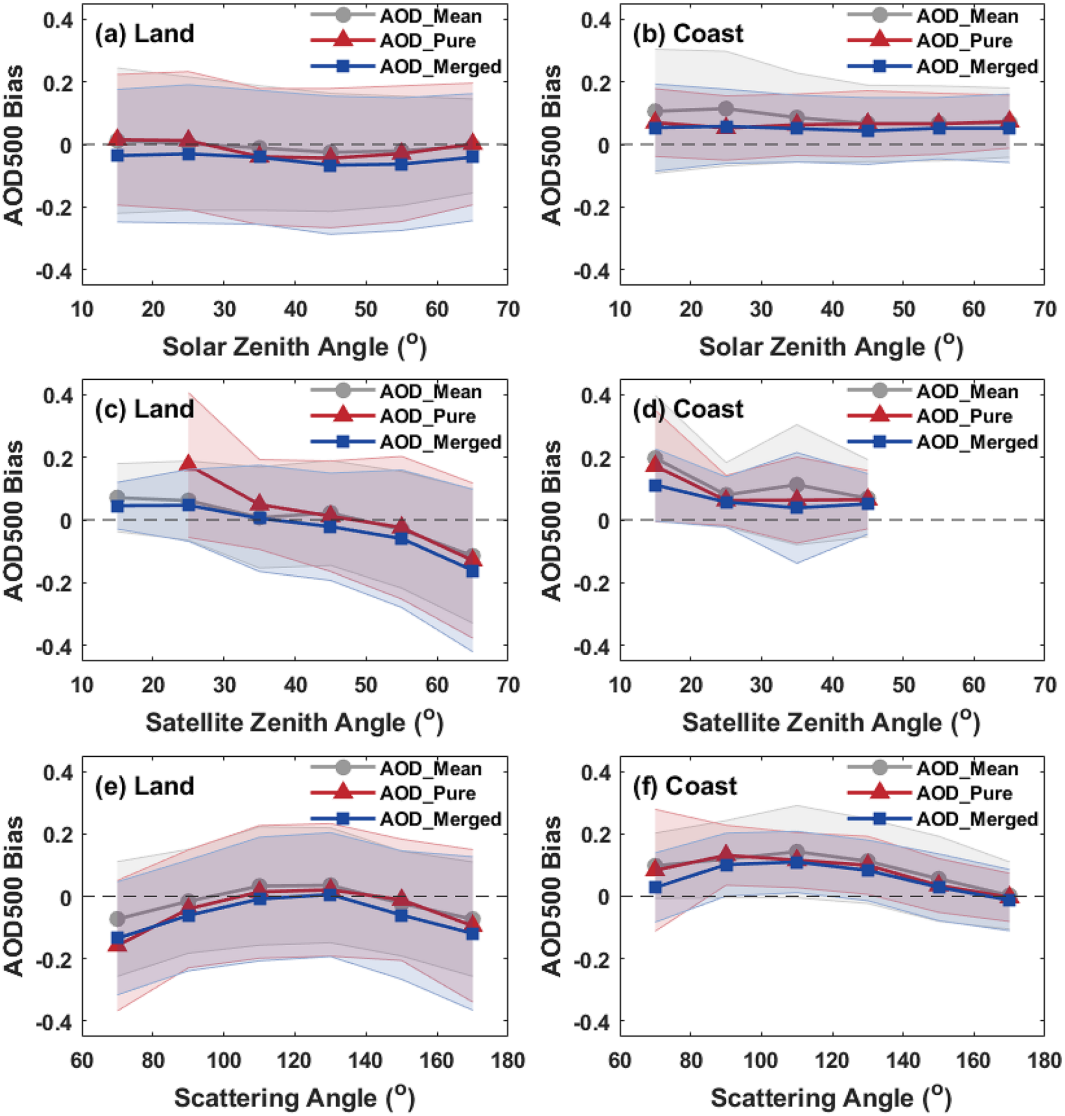
The dependence of AOD retrieval bias (AHI AOD-AERONET AOD) with solar zenith angle, satellite zenith angle and scattering angle for different AHI L3 AOD datasets (AOD_mean_, AOD_pure_ and AOD_merged_) over land (**a**,**c**,**e**) and coast (**b**,**d**,**f**).

To further examine the geometry dependency of different AHI aerosol products, the AOD retrieval bias in AHI products with solar zenith angle, satellite zenith angle and scattering angle is shown in Fig. 9. As shown, the accuracy of AHI AOD retrievals are less dependent on solar zenith angle in the geometry dependency analysis. In Fig. 9c, the AOD bias changes from positive (~ 0.05) to negative (~ − 0.15) with the increase of satellite zenith angle and a relatively large bias of AOD_pure_ in the range of 20° to 30° is due to the less matchups in this case. Over coast, there is an obvious missing of AHI AOD matchups when the satellite zenith angle is larger than 50° and a relatively high retrieval error in AHI AOD retrievals can be found in the small satellite zenith angle (10°–20°). Figure 9e,f show that the relation between AHI AOD bias and scattering angle follows an inverted U-shaped curve. The AHI AOD retrievals are severely underestimated over land when scattering angle is larger than 160° or smaller than 80°, but with the optimum performance in the range of 100° to 140°. Furthermore, the coastal AOD can be retrieved more accurately at a larger (> 140°) or smaller (< 80°) scattering angle. The scattering angle which affects the scattering phase function in radiative transfer model varied with time is considered to be one of the important factors that results in AHI temporal variations. Overall, the AHI AOD_mean_, AOD_pure_, and AOD_merged_ retrievals exhibit a similar bias trend with the variation of solar zenith angle, satellite zenith angle and scattering angle.

### Temporal variation evaluation with AERONET

**Figure 10:**
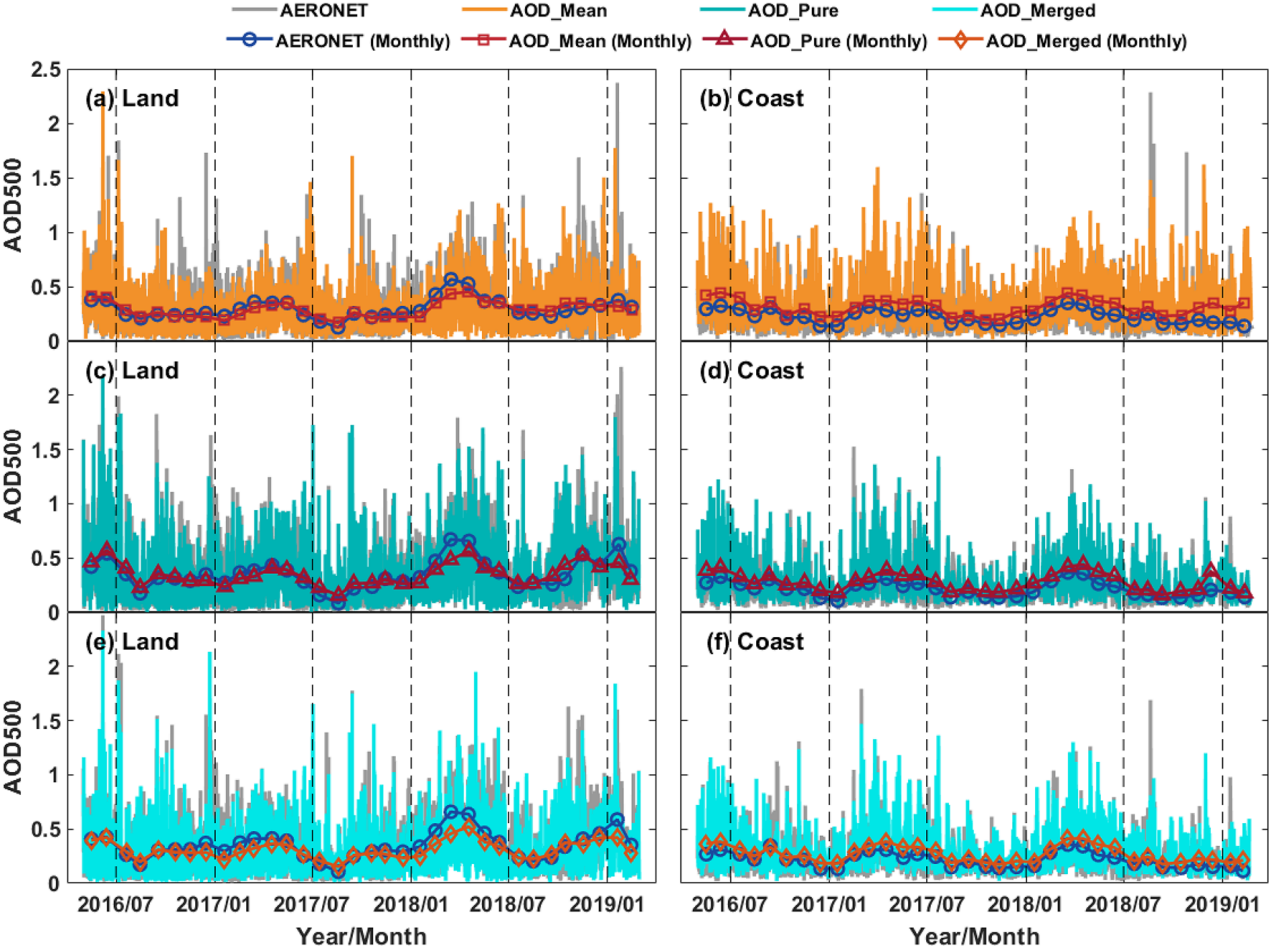
Time sequences of the hourly (solid lines) and monthly (solid lines with marker) AOD from AHI and AERONET observations over land and coast from May 2016 to February 2019. The hourly and monthly AHI AOD_mean_ over land (**a**) and coast (**b**) are indicated by the solid orange and red lines with square markers, respectively. The hourly and monthly AHI AOD_pure_ over land (**c**) and coast (**d**) are indicated by the solid dark cyan lines and dark red lines with upward-pointing triangle markers, respectively. The hourly and monthly AHI AOD_merged_ over land (**e**) and coast (**f**) are indicated by the solid cyan lines and dark orange lines with diamond markers, respectively. The hourly and monthly AERONET AOD in all panels are indicated by the gray solid lines and blue solid lines with circle markers, respectively.

Figure 10 presents the time series of hourly and monthly averaged AOD of AHI and AERONET over land and coast between May 2016 to February 2019. Over land, the relatively large biases of AHI AOD observations appear in December to March, which may be related to the frequent haze events and in northern hemisphere during this period^32^. Meanwhile, the coastal AHI AOD observations are generally overestimated throughout the period of the study, as shown in the right panel of Fig. 10. Besides, the relatively large biases of AHI AOD relative to ground-based AOD can be found from November 2018 to February 2019 in Fig. 10b. But remarkably, there are more AOD_mean_ matchups falling around the AERONET sites located on the Australian coast (Lucinda: 146.386°E, 18.520°S) and near the equator (Sorong: 131.268°E, 0.875°S; Pontianak: 109.191°E, 0.075°N; Penang: 100.302°E, 5.358°N) during this period, which has been proved to be highly overestimated in Fig. 3g–i. Overall, the temporal results demonstrate that AHI AOD products show good consistency with AERONET.

### Evaluation for land surface cover

**Figure 11:**
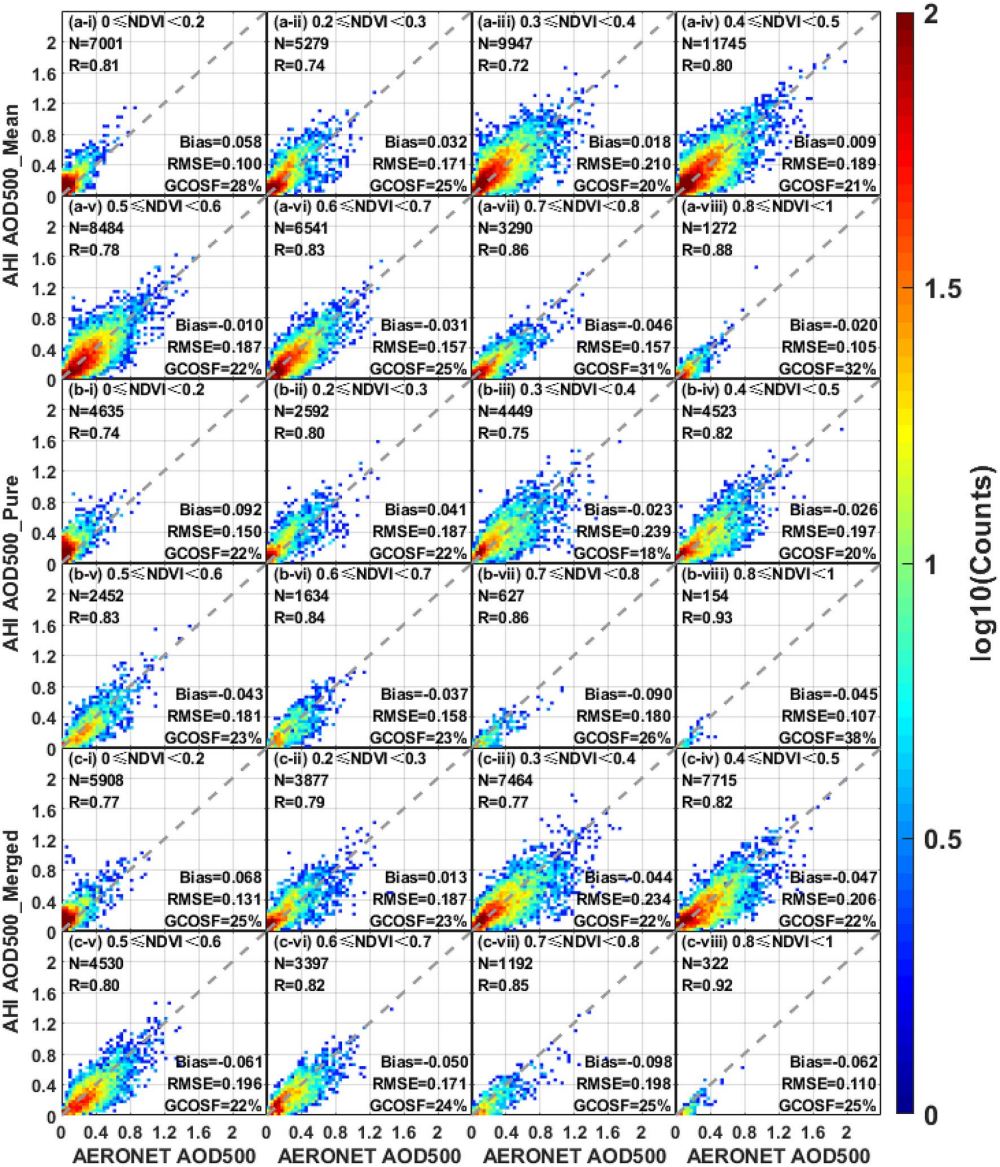
Scatter plots of AHI AOD versus AERONET observations over land surface with different vegetation cover. The panels (**a**–**c**) represent different AHI L3 AOD datasets (AOD_mean_, AOD_pure_ and AOD_merged_), respectively. The subplots (i–viii) indicate the NDVI values from 0 to 1: (i) 0–0.2, (ii) 0.2–0.3, (iii) 0.3–0.4, (iv) 0.4–0.5, (v) 0.5–0.6, (vi) 0.6–0.7, (vii) 0.7–0.8 and (viii) 0.8–1.

Different land cover types affect satellite-derived aerosol product by influencing surface reflectance estimation. Figure 11 displays the scatterplots of AHI aerosol products against AERONET measurements under different NDVI ranges. Overall, AHI AOD retrievals show a better performance over dense vegetation areas (NDVI ≥ 0.7) than bright surfaces (NDVI < 0.3), with a higher R (0.85–0.93 vs. 0.74–0.81) and GCOSF percentage (25–38% vs. 22–28%), and all the AHI retrieval biases change from positive to negative with the increase of NDVI values. Moreover, when NDVI ≥ 0.6, AHI AOD_mean_ retrievals perform better against AERONET AOD than AOD_pure_ and AOD_merged_ products, with a lower RMSE (0.105–0.157 vs. 0.107–0.180 and 0.110–0.198) and a smaller bias (− 0.020 to − 0.046 vs. − 0.037 to − 0.090 and − 0.050 to − 0.098). This suggests that the AHI hourly combined algorithm may erroneously eliminate many normal observations and cause a serious underestimation over these regions.

### Evaluation for meteorological conditions

**Figure 12:**
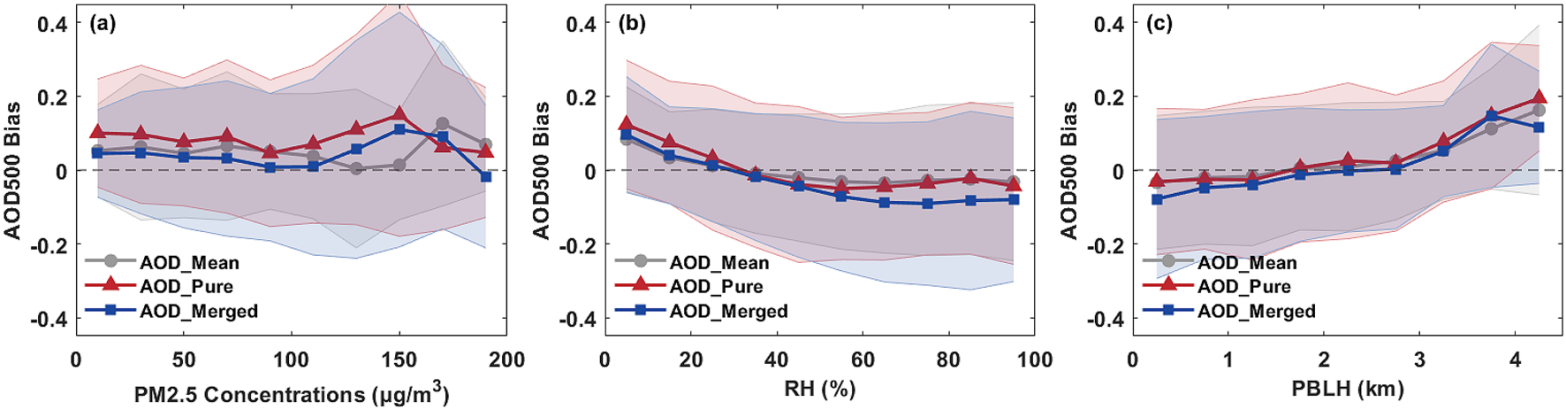
Validation of AHI AOD retrievals with AERONET observations under different meteorological conditions: (**a**) PM_2.5_ concentrations; (**b**) Relative humidity (RH); (**c**) Planetary boundary layer height (PBLH).

To evaluate the performance of AHI aerosol products under different air pollution levels, Fig. 12a demonstrates the relation of AHI retrieval biases (AHI AOD-AERONET AOD) and ground PM_2.5_ concentration. It is worth noting that AHI can retrieve aerosol with a stable accuracy at low pollution levels. However, AHI AOD_pure_ and AOD_merged_ retrievals are significantly overestimated when PM_2.5_ concentration is higher than 110 μg/m^3^, while AOD_mean_ still presents a relatively smaller bias within the PM_2.5_ concentration range of 110–150 μg/m^3^. For the heavy pollution level (> 150 μg/m^3^), AOD_pure_ and AOD_merged_ retrievals achieve a better agreement with AERONET than AOD_mean_. Therefore, the hourly combined algorithm is more susceptible to haze events under the moderate pollution level (115–150 μg/m^3^).

Relative humidity (RH) is an important proxy to describe the water vapor content in atmosphere, and the water vapor content play a role in aerosol components and properties. As shown in Fig. 12b, AHI AOD is overestimated at a low relative humidity (RH < 20%) and underestimated when relative humidity is higher than 40%. The optimum humidity condition for AHI AOD retrieval is within the range of 20–40%. Moreover, more severely underestimated retrievals can be found in AOD_merged_ under moist atmosphere (RH > 60%), while AHI AOD_pure_ exhibits a relatively high positive bias for dry atmosphere (RH < 20%). The PBLH is another important meteorological parameter to characterize the lowest part of atmosphere. Thus, Fig. 12c displays the effect of PBLH on different AHI AOD retrievals. Obviously, all the AHI AOD retrievals are weakly dependent on the PBLH when it is lower than 3 km. But for a higher PBL, the biases of AHI AOD retrievals increased quickly with the height. In addition, there are no significant differences in retrieval accuracy among the AHI AOD_mean_, AOD_pure_ and AOD_merged_ products in Fig. 12c, which suggests that the PBLH has little contribution to the performance discrepancies of the three AOD retrievals.

### Regional evaluation with MAN

**Figure 13:**
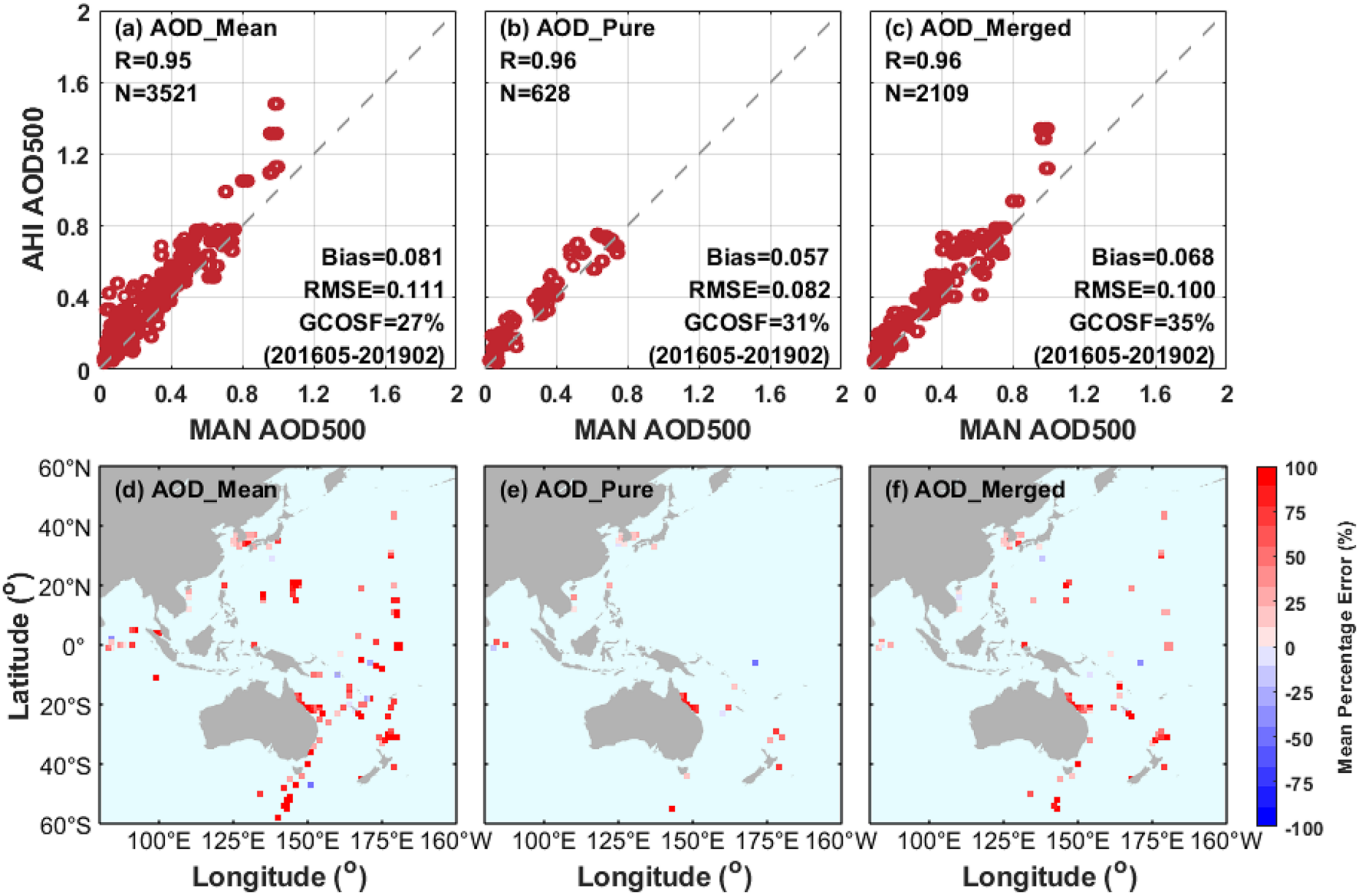
Validation of AHI AOD aerosol retrievals with MAN observations over ocean from May 2016 to February 2019. (**a**–**c**) Scatter plots of AHI AOD retrievals versus MAN observations. (**d**–**f**) Mean percentage error of AHI AOD retrievals relative to the MAN observations at each 1° × 1° grid. The validation results of different AHI L3 AOD datasets (AOD_mean_, AOD_pure_ and AOD_merged_) are listed in columns 1, 2 and 3, respectively. The figure is generated by the software package M_Map^39^ (version 1.4 m; www.eoas.ubc.ca/~rich/map.html).

The comparison results of all AHI L3 AOD retrievals against ship-borne AOD measurements for the open ocean regions are depicted in Fig. 13 to supplement the results for AERONET coastal sites. The scatter plots of the AHI AOD against MAN AOD show that the retrieval accuracy in AOD_merged_ is better compared with AOD_mean_, with a lower bias (0.068 vs. 0.081), a smaller RMSE (0.100 vs. 0.111), and a larger GCOSF (35% vs. 27%). Besides, the AOD_pure_ shows slightly better agreement with MAN observations in comparison to AOD_merged_, with a lower bias of 0.057 and a smaller RMSE of 0.082. As for the performance of AHI aerosol products at regional scales, the retrievals are generally overestimated in most ocean areas but relatively slightly overestimated in the oceans around South Korea and Japan. Contrary to the AHI land retrievals in Fig. 2, AOD_mean_ retrievals without strict cloud screening show poorer performance than AOD_pure_ and AOD_merged_, exhibiting high positive biases (~ 100%) in most of the sites over oceanic regions. The spatial variability of AOD is considered to be greater over land than over ocean^30^, but the same spatial window is used for hourly combined retrievals (AOD_pure_ and AOD_merged_) over both land and ocean. This may lead to the performance discrepancies of the AOD_pure_ and AOD_merged_ retrievals between land and ocean when compared to the AOD_mean_ retrievals, and it is probably better to use a smaller spatial window for the land retrievals in the hourly combined algorithm. Moreover, there are relatively fewer positive biases observed in AOD_pure_ compared with AOD_merged_ retrievals, although AOD_pure_ still with high positive biases against MAN observations over the South Pacific. However, AHI ocean retrievals are affected by other factors such as the edge artifacts of the sun-glint mask and terminator line, which deserves further research and exploration^53^.

### Comparison with MODIS

**Figure 14:**
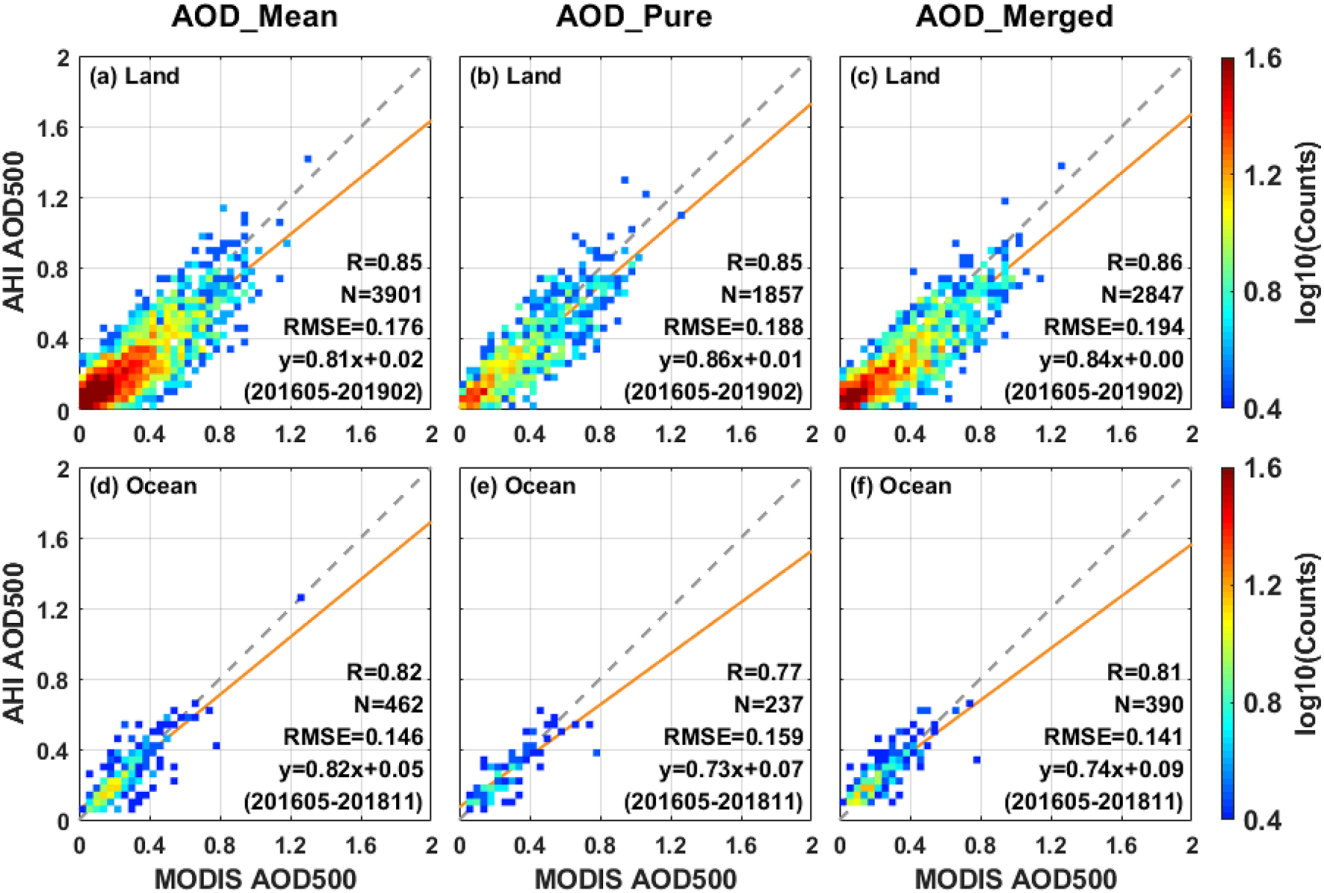
Density scatter plots of AHI AOD against MODIS AOD over land (**a**–**c**) and ocean (**d**–**f**) from May 2016 to February 2019. The 1:1 lines and linear regression lines of scatter plots are plotted as gray and orange lines. The comparison results of different AHI L3 AOD datasets (AOD_mean_, AOD_pure_ and AOD_merged_) are listed in columns 1, 2 and 3, respectively.

To provide an additional independent measurement other than AERONET, the evaluations of AHI against MODIS are also included in this study. The scatter plots of AHI AOD against MODIS AOD over the land and ocean against MODIS AOD are presented in Fig. 14. The comparison results demonstrate that there is a similar linear relationship between different AHI products and MODIS retrievals over land, with an approximate slope (0.81–0.86) and intercept (0–0.02). The AHI products tend to underestimate AOD compared with MODIS retrievals over land, which suggests that the AOD retrieval performance of Himawari-8/AHI remains to be further improved and needs to narrow the gap with MODIS collections. Over ocean, the linear fitting slope of AOD_mean_ product against MODIS product is closer to the 1:1 line than that of AOD_pure_ and AOD_merged_ (0.82 vs. 0.73 and 0.74). The performance differences between AHI AOD and MODIS AOD may depend on their own unique algorithms, observation geometries and sensor characteristics^36,38^. However, there may be larger positive deviations observed in MODIS retrievals over ocean, because the slopes of AHI AOD against MODIS AOD are lower than 1 in Fig. 14d–f and AHI retrievals present a high positive bias over ocean in Fig. 13. For the configuration of spectral bands, AHI lacks a 1.38 µm channel for the cirrus cloud detection compared to MODIS, which may cause the differences of cloud recognition before AOD retrieve.

### Evaluation of spatio-temporal coverage

**Figure 15:**
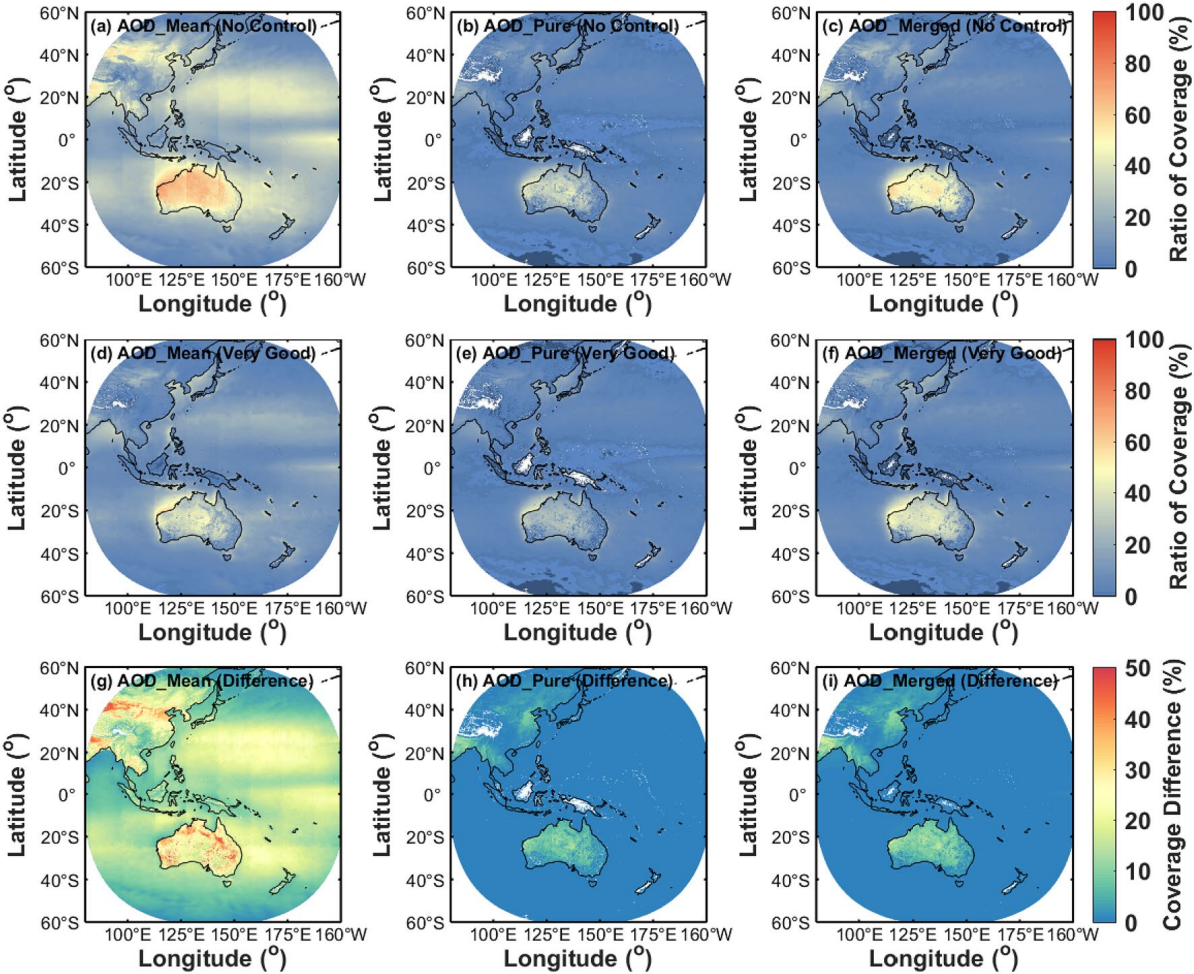
Validation of spatial coverage AOD for different AHI L3 AOD datasets (AOD_mean_, AOD_pure_ and AOD_merged_) over land and ocean from May 2016 to February 2019. (**a**–**c**) The coverage ratio of all available AHI retrievals without quality control. (**d**–**f**) The coverage ratio of AHI retrievals with QA screening (QA flag = very good). (**g**–**f**) The coverage differences of AHI retrievals before and after QA screening. The figure is generated by the software package M_Map^39^ (version 1.4 m; www.eoas.ubc.ca/~rich/map.html).

The spatial coverage ratio from May 2016 to February 2019 is evaluated between different AHI hourly aerosol products in Fig. 15. As illustrated in Fig. 15a–c without QA screening, the AOD_pure_ and AOD_merged_ products have much lower spatial coverage than the AOD_mean_ products over most land and ocean regions due to the removal of cloud-contaminated observations, especially in the Qinghai-Tibet Plateau, New Zealand, and tropical islands such as Borneo and New Guinea. These missing values are mainly for the equatorial regions with more clouds and the high-altitude areas covered by snow and ice, where most of the AHI retrieval algorithms cannot be implemented. Furthermore, AOD_merged_ can be obtained by interpolating if there are sufficient AOD_pure_ observations around^29,30^. Therefore, it can be found in Fig. 15b,c that the coverage ratio of AOD_pure_ has been recovered to some extent in AOD_merged_.

To evaluate the impact of QA levels on the coverage of different AHI AOD products, Fig. 15d–i show the influence of different quality controls on the spatial coverage of AHI hourly products in Himawari-8 observation regions. Considering the QA flags of “good” and “marginal” are not used in the AHI datasets, only the AOD retrievals with the QA confidence level of “very good” are discussed in this study. As presented in Fig. 15d–f, there is a comparatively higher coverage in AOD_mean_ than products after the QA screening, and the highest percentage of AHI observations is also caught in Australia than other regions. For the differences in Fig. 15g–i, the variation of AOD_mean_ is found to be much larger than AOD_pure_ and AOD_merged_, with a percentage of > 20% in most areas. Moreover, there is a lower percentage of < 5% for the coverage changes over all the oceanic and most terrestrial regions in AOD_pure_ and AOD_merged_ retrievals (Fig. 15h,i). Moreover, the relatively large coverage variations of AHI products can be observed in northern China, eastern India, Myanmar, Thailand and Australia, which probably mean a larger uncertainty of AHI retrieval algorithm in these areas.

## Conclusions

In this study, the latest Himawari-8/AHI hourly atmospheric aerosol product and simple customized product were validated by AERONET and MAN observations over land and ocean in the Himawari-scope region from May 2016 to February 2019. The extended comparisons between different datasets of AHI aerosol products (i.e., AOD_mean_, AOD_pure_ and AOD_merged_) and the difference in performance of satellite-derived retrievals between AHI and MODIS were discussed in detail. Over land, the matched AOD_mean_ retrievals showed a slightly better performance compared with AOD_pure_ and AOD_merged_ products and capture a larger number of coincident observations in the same spatiotemporal window. For the regional comparisons, AHI AOD products achieved a relatively better performance in East Asia, whereas the significant regional underestimations and overestimations can be observed in Southeast Asia and Australia, respectively. Over coast, the AHI AOD_merged_ was generally agree better with the AERONET AOD than AOD_mean_ and AOD_pure_ products and AHI retrievals presented a comparatively smaller uncertainty at the coastal sites of East Asia. Over ocean, it can be found that the AOD_pure_ achieved a better agreement with AERONET AOD as compared to that of AOD_mean_ and AOD_merged_ retrievals. However, overall AHI ocean observations were extensively overestimated by the validation with ship-borne AOD measurements, especially showing a large number of high positive biases (~ 100%) over the South Pacific. For the L3 AE products, AE_mean_ and AE_merged_ provided more accurate retrievals than AE_merged_, and exhibited similar performance over both land and coast.

Comparing the results under different daytime hours, the best performance of AHI AOD retrievals occurred in the late afternoon (16:00–17:00 LT) over land and around the noon (10:00–13:00 LT) over coast, respectively, which is mainly affected by the scattering angle. AHI AOD retrieval accuracy can be also influenced by many other factors, such as surface NDVI, PM_2.5_ concentrations, RH, PBLH, etc. The comparison results indicated a good correlation between AHI and MODIS, but AHI products generally underestimated AOD relative to MODIS retrievals over land and ocean. For the spatial coverage, more observations were available in AOD_mean_ over Himawari-domain region, with a higher percentage of coverage than AOD_pure_ and AOD_merged_. Moreover, it should be noted that AHI AOD retrievals lack adequate observations in the Qinghai-Tibet Plateau, New Zealand, and tropical islands such as Borneo and New Guinea.

This work has evaluated different Himawari-8/AHI hourly aerosol products in version 3.0, and we suggest that the regional performance differences and data availability should be taken into consideration in the selection of AHI products in related aerosol studies. The underlying mechanisms of Himawari-8/AHI AOD performance differences under various influential factors must be further analyzed in future research, and AHI retrieval algorithm requires further improvement to highlight its advantage of high temporal resolution.

## Data Availability

The datasets generated and analyzed during the current study are available from the corresponding author on reasonable request.
